# Enhancement of HIFU thermal therapy in perfused tissue models using micron-sized FTAC-stabilized PFOB-core endovascular sonosensitizers

**DOI:** 10.1080/02656736.2020.1817575

**Published:** 2020-09-29

**Authors:** Orane Lorton, Pauline C. Guillemin, Ryan Holman, Stéphane Desgranges, Laura Gui, Lindsey A. Crowe, Sylvain Terraz, Antonio Nastasi, François Lazeyras, Christiane Contino-Pépin, Rares Salomir

**Affiliations:** a Image Guided Interventions Laboratory (GR-949), Faculty of Medicine, University of Geneva, Geneva, Switzerland; b University of Avignon, CBSA-IBMM, (UMR5247), Avignon, France; c Radiology Department, University Hospitals of Geneva, Geneva, Switzerland; d Visceral and Transplantation Division, University Hospitals, Geneva, Switzerland; e Center for Biomedical Imaging (CIBM), Geneva, Switzerland

**Keywords:** MRgHIFU, sonosensitizers, micro-droplets, perfused model, thermal deposition

## Abstract

**Background:**

High intensity focused ultrasound (HIFU) is clinically accepted for the treatment of solid tumors but remains challenging in highly perfused tissue due to the heat sink effect. Endovascular liquid-core sonosensitizers have been previously suggested to enhance the thermal energy deposition at the focal area and to lower the near-/far-field heating. We are investigating the therapeutic potential of PFOB-FTAC micro-droplets in a perfused tissue-mimicking model and postmortem excised organs.

**Method:**

A custom-made *in vitro* perfused tissue-mimicking model, freshly excised pig kidneys (*n* = 3) and liver (*n* = 1) were perfused and subjected to focused ultrasound generated by an MR-compatible HIFU transducer. PFOB-FTAC sonosensitizers were injected in the perfusion fluid up to 0.235% v/v ratio. Targeting and on-line PRFS thermometry were performed on a 3 T MR scanner. Assessment of the fluid perfusion was performed with pulsed color Doppler *in vitro* and with dynamic contrast-enhanced (DCE)-MRI in excised organs.

**Results:**

Our *in vitro* model of perfused tissue demonstrated re-usability. Sonosensitizer concentration and perfusion rate were tunable *in situ*. Differential heating under equivalent HIFU sonications demonstrated a dramatic improvement in the thermal deposition due to the sonosensitizers activity. Typically, the energy deposition was multiplied by a factor between 2.5 and 3 in perfused organs after the administration of micro-droplets, while DCE-MRI indicated an effective perfusion.

**Conclusion:**

The current PFOB-FTAC micro-droplet sonosensitizers provided a large and sustained enhancement of the HIFU thermal deposition at the focal area, suggesting solutions for less technological constraints, lower risk for the near-/far- field heating. We also report a suitable experimental model for other MRgHIFU studies

## 1. Background

High intensity focused ultrasound (HIFU) is a noninvasive and non-ionizing technique that has been widely accepted for ablation of solid tumors such as prostate tumors, breast cancer or uterine fibroids [1–6]. Such ablation leads to coagulative necrosis or cellular apoptosis [7]. Additionally, HIFU treatments can be combined with magnetic resonance imaging monitoring (MRgHIFU) due to the excellent soft tissue contrast of MRI, allowing treatment planning and thermometry with real-time temperature monitoring based on proton resonance frequency shift (PRFS) method [8].

Moreover, the MRgHIFU technique may be used as an adjuvant in cancer treatment such as radiation therapy or chemotherapy by delivering localized and mild heating (41–43 °C) over 30–60 min [9,10] to increase the cell sensitivity [11]. Elevating temperature above the physiological level shows a complementary action when used in conjunction with radiation therapy with regard to cell killing by increasing cell radiosensitivity, improving reoxygenation [12] and inhibiting crucial DNA repair pathways [13–15]. In addition, mild hyperthermia may also be combined with chemotherapy to prevent tumor progression, reduce the tumor size and improve survival in patients [9].

However, MRgHIFU in highly perfused tumors is challenging because higher energy is required to compensate for the cooling of the tissues induced by blood flow, called the heat sink effect [16]. Subsequently, critical side effects are produced, such as skin or bone burning, post focal energy deposition or side lobe effects [17–19]. Jung et al. [20] reported a significant number of adverse effects occurring after HIFU treatment of hepatic tumors, hepatic metastasis and pancreatic cancers such as skin burning, edema, and, in all patients, pain. Even more severe, necrosis of the ribs along the beam path appeared in all patients with hepatic tumor, which may lead to rib fracture in long-term. Several other complications outside the target region were observed including symptomatic pleural effusion, pneumothorax and third-degree skin burns. The size of ablative lesion is also limited and the treatment time is increased, ranging from 3.5 to 7.5 h depending on the cancer type according to Jung et al. [20]. Quantitatively, high energy is required to reach sufficient temperature (up to 6800 J per sonication in Napoli et al. [21], up to 3645 J per sonication in Geiger et al. [22] and up to 3522 J per sonication in Rovella et al. [23]), increasing chances of undesirable side effects.

When targeting perfused tissue with HIFU, therefore persists a stringent need to improve the thermal contrast between the focal area and the near- and far-field. Some methods to alleviate near- or far-field side effects have been reported such as shielding the rib cage [24]. This consists of placing an absorbent and/or reflective layer in front of the rib cage to stop the HIFU beams oriented on the ribs. Another method to protect the rib cage was reported by Quesson et al. [25] and consists in selecting the focal area, manually segmenting the ribs on MR images, projecting the segmented area on the transducer surface according to the beam path and deactivating elements of the HIFU transducer susceptible to burn the rib cage. Non-linear effects such as shock waves were also investigated for increased energy absorption using a pulsed ablation strategy with 1 kW peak power [26]. This approach enabled an ablation rate on the order of 4 ml/h without noticeable side effects in the near field. The localized non-linear phenomena are expected, however, to be tissue dependent. Overall, while the reported extracorporeal techniques have showed noticeable improvement, they run into some limitations and more effective approaches are still needed.

Besides the high blood perfusion in liver and kidney, their treatment by HIFU is further complicated by breathing-related motion [27,28]. Accurate compensation of tissue motion was demonstrated by various authors; however, most of the existing approaches also reduce the effective energy deposition at the focus. Temporal gating of sonication during the quiet phase of respiration obviously reduces the duty cycle [29,30] and yields a long duration of the procedure. When motion tracking is performed with electronic steering using a phased array applicator, lateral focusing is less effective than natural focus sonication [31–35].

To increase the thermal efficiency of HIFU and subsequently to reduce side effects, introducing gas-filled bubbles into the targeted region was suggested [36,37]. Lipid-shelled microbubbles (MBs) filled with perfluorocarbon (PFC) gas, primarily developed as an ultrasound (US) contrast agent, were used as enhancers of HIFU thermal ablation. MBs exposed to HIFU will undergo nonlinear oscillation known as inertial cavitation (IC) that will radiate energy into the surrounding tissue. The acoustic pressures required to generate inertial cavitation *in vivo* are generally high and tissue-specific [38]. MBs can act as cavitation nuclei in a targeted area lowering cavitation thresholds and increasing the rate of tissue heating during HIFU treatment. Despite their clinical success, MBs exhibit two major limitations, firstly their short *in vivo* half-life (in order of minutes), and secondly, regarding HIFU ablation therapy, significant heating and damage were observed along the HIFU beam, as they can be activated throughout a low intensity acoustic field due to their very low IC threshold [39,40].

In order to overcome these limitations of MBs, a second generation of HIFU sensitizers was developed based on a process called acoustic droplet vaporization (ADV) [41]. This concept involves phase shift droplets (PSD) filled with a liquid PFC, that can be in a superheated state, and have the capacity to be vaporized into microbubbles under US stimulation [42]. ADV has been used by several groups with two different classes of droplets [43,44]. The first one includes phase-shift nanodroplets (PSND), that can passively accumulate in tumors by means of the so-called Enhanced Permeability and Retention (EPR) effect [45,46]. The bubbles generated from PSND by acoustic stimuli can serve as ultrasound contrast agents, drug delivery systems or HIFU sensitizers [47–53]. The second one targets endovascular applications with phase-shift micron-sized droplets (PSMSD). According to their mean diameter PSMSD can be used for embolotherapy as they generate micro-bubbles able to occlude small capillary vessels, for sonothromobolysis [41,54] as well as for HIFU driven thermal ablation of highly perfused tumors given that PSMSD circulate only within the tumor microvasculature [55,56].

According to Kopechek et al., nanodroplets in the size range of 150–200 nm have the advantage to accumulate by the EPR effect in tumors, and the pool of nanoparticles were used as sonosensitizers at one time-point after several hours of ‘natural’ accumulation period [43]. Contrarily, after injection of high boiling point endovascular micron-sized droplets, a constant and uniform concentration should be immediately available in the vascular pool and the response to HIFU should be steady/repeatable over the time, for the duration of the ablative procedure. This is suggested to be a fundamental advantage in term of treatment planning and intraoperatory control.

In a previous study, Desgranges et al. [57] reported the use of perfluorooctylbromide (PFOB)-filled micron-sized droplets as a new class of sonosensitizers for enhanced HIFU thermal therapy. PFOB is FDA approved and is used for its high oxygen solubility, low toxicity and short half-life time in the body, making it the best candidate to be used as a contrast agent in human tissues [58]. These PFOB sonosensitizers are composed of liquid-core PFOB with a higher boiling point (142 °C) compared to other PFC. They were stabilized with a biocompatible in-house synthesized F-TAC fluorinated surfactant. Among the F-TAC family of molecules, the specific surfactant was chosen according to the desired droplet size, in the range 0.67 µm–4.07 µm mean diameter. The mean diameter range 1–4 µm is considered optimal in order to 1) circulate inside the endovascular system avoiding embolism and 2) avoid short term leaking outside the vessels. A semi-rigid tissue-mimicking gel was homogeneously doped with 0.1%–0.5% of PFOB-filled sonosensitizers, demonstrating a significant enhancement of HIFU absorption. Importantly, using a sonosensitizer model immobilized in a semi-rigid matrix gel, the HIFU beam did not alter the micron-sized droplet’s capability to enhance the ultrasound absorption for the next sonication repetition. The micron-sized droplets being immobilized in the semi-rigid gel matrix could be sonicated repeatedly without significant loss of efficacy.

Nevertheless, the mentioned study was performed in tissue-mimicking gels with trapped sonosensitizers at volume concentrations too high for the direct translation to *in vivo*. The main goals of the current study were 1) to develop a new formulation of liquid-core PFOB-FTAC micro-droplets that are sufficiently stable in liquid emulsions for endovascular circulation, 2) to demonstrate the enhancement of the HIFU thermal effect in *in vitro* tissue-mimicking perfused phantoms, 3) to demonstrate the enhancement of the HIFU thermal effect after injection of endovascular sonosensitizers in the perfusion fluid of freshly excised pig kidney and liver and 4) to demonstrate an effective circulation of sonosensitizers in perfused *ex vivo* organs proving no embolism was created. MR imaging was used for intra-operative targeting and PRFS thermometry, as well as assessing the perfusion in freshly excised organs.

## 2. Material & method

### 2.1. Sonosensitizers

**Figure 1.**
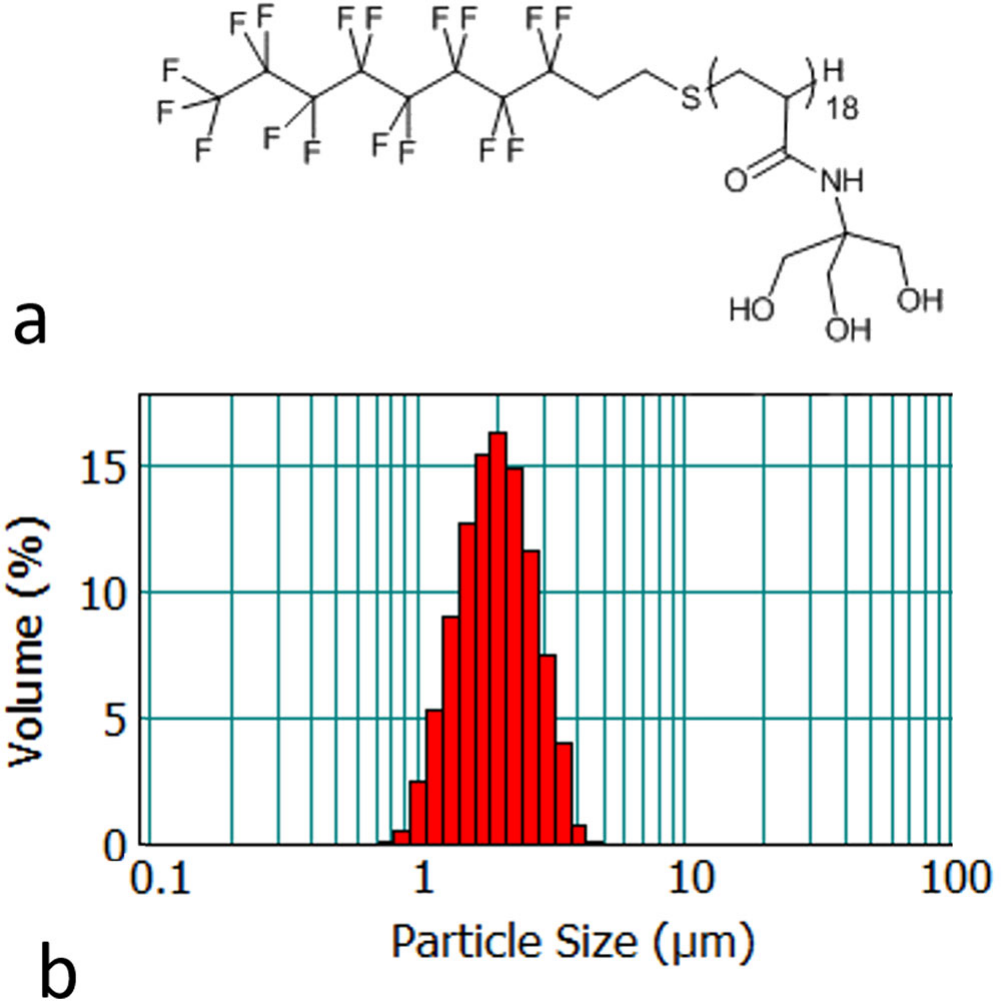
(a) Illustration of the structure of the F_8_TAC_18_ surfactant and (b) illustrative histogram of F_8_TAC_18_-PFOB sonosensitizer size distribution, provided as volume fraction.

The surfactant F_8_TAC_18_ was chosen because it yielded the required size of sonosensitizers for intra-capillary circulation and assured sufficient stability of micro-droplets in a liquid emulsion. F-TAC molecules consist of a fluorinated hydrophobic moiety exhibiting a high affinity for PFC droplets and a hydrophilic moiety made of a polyTRIS oligomer as illustrated in Figure 1(a). The surfactant was synthesized in one step by free radical polymerization as previously described [57]. The liquid-core PFOB-FTAC sonosensitizers were prepared using a Polytron homogenizer (model PT3100) from Kinematica (Luzern Switzerland). Namely, to prepare 220 ml of an emulsion at 2% volume fraction, 550 mg of F_8_TAC_18_ surfactant were dissolved in 215.6 ml of water and then 4.4 ml of PFOB were added. The resulting mixture was cooled down with an ice bath and the resulting emulsion was homogenized three times, each for a duration of 15 min, at 20,000 rpm. Unlike the previous study [57], which encapsulated the micro-droplets in a semi-rigid gel, stable fluid emulsions of sonosensitizers were prepared with 2% v/v of PFOB and purified water. The emulsion was kept at 4 °C until use and was stable for at least one month.

The particle size distribution (Figure 1(b)) was assessed using a Mastersizer 2000 laser diffraction particle size analyzer (Malvern Instruments, Orsay, France) equipped with Hydro2000S as the sample dispersion unit. Mie theory was used to determine the volume weighted mean diameter [3,4]. The refractive indices used were 1.305 for the PFOB and 1.333 for the dispersant (water). Several drops of the emulsion were added to the sample dispersion unit with 1855 rpm stirring.

### 2.2. Perfused tissue-mimicking model

**Figure 2.**
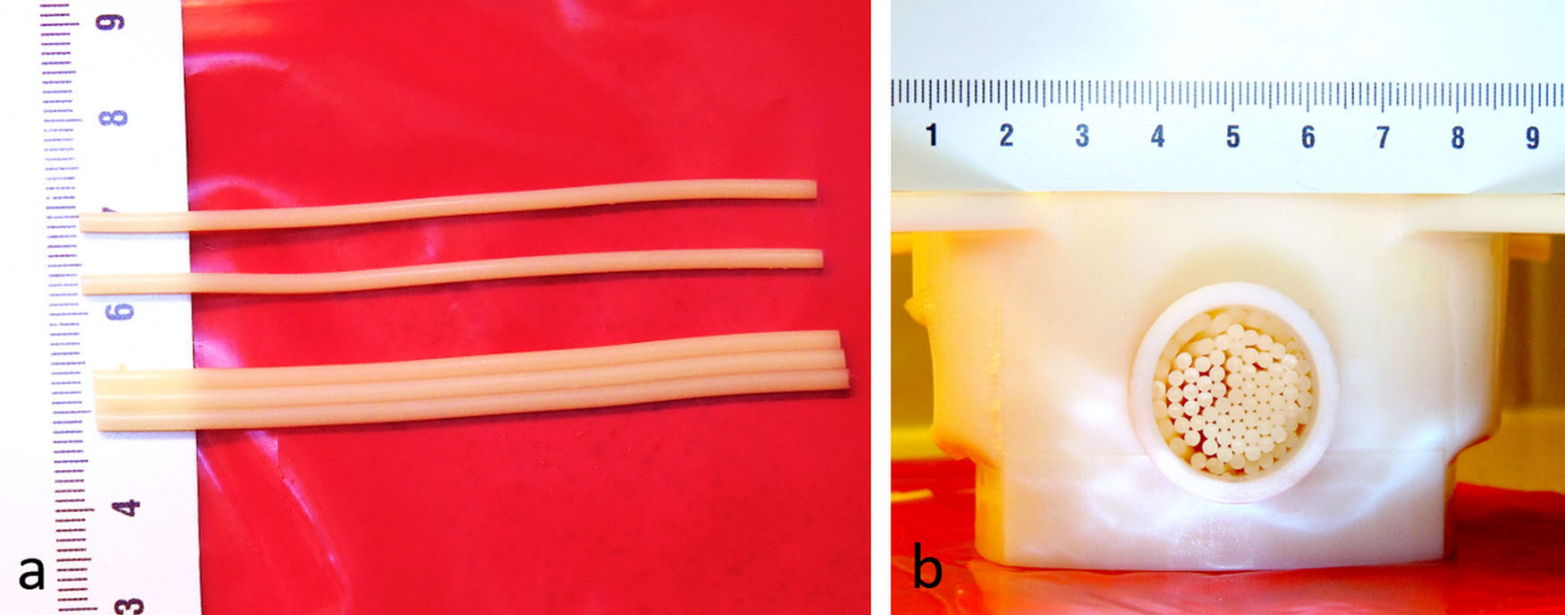
(a) Shaping of individual gel strands composed of tissue-mimicking gel (agar, glycerin, evaporated milk and degassed water). (b) Front view of the in vitro model of perfused tissue composed of densely packed gel strands embedded in a cylindrical cavity.

**Figure 3.**
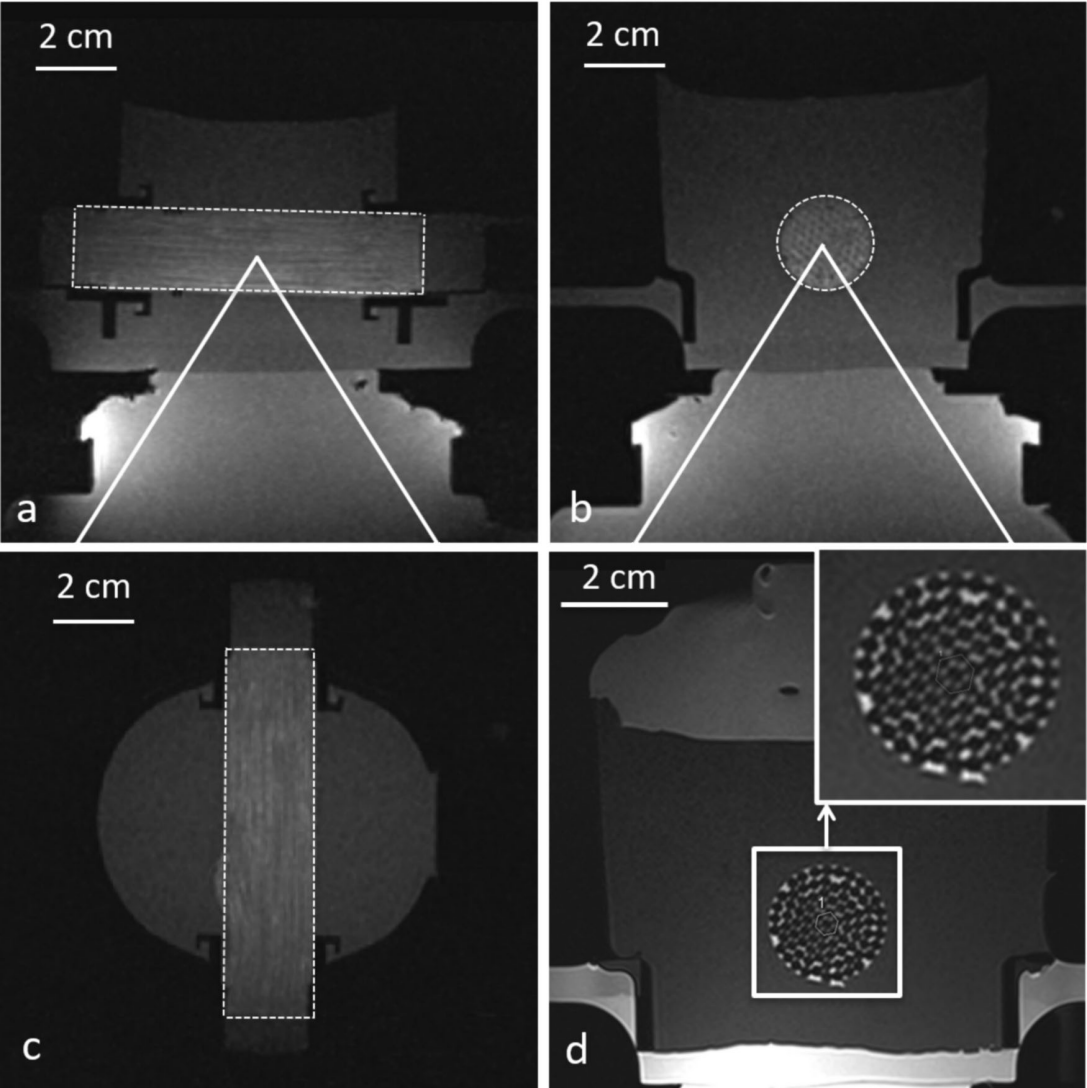
T1-VIBE high resolution MR images of the in vitro perfused model in three orthogonal planes (a) transverse, (b) sagittal and (c) coronal. T2-TSE high resolution MR image in the transverse (d), demonstrating the hexagonal packing with a gel-filling volume ratio of approximately 90% inside the cylindrical cavity of the matrix gel.

**Figure 4.**
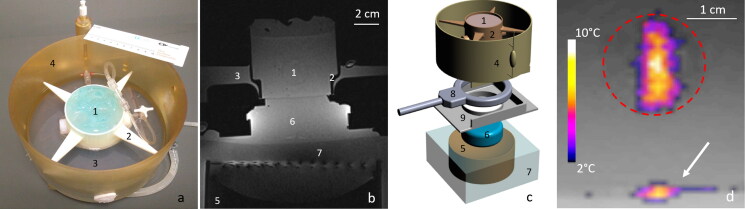
Insights of the in vitro model of perfused tissue hardware and monitoring of MRgHIFU therapy. (a) Photograph of the in vitro model embedded in its dedicated holder, designed to avoid vibrations, and locked in the MR-compatible perfusion machine, (b) T1-weighted MR transverse image of the full set up matched with the 3D drawing of the different elements shown in frame (c). Legend component parts: 1. In vitro model of perfused tissue covered by standard ultrasonic gel, 2. 3D printed holder, 3. Perfusate, 4. Perfusion circuit and reservoir, 5. HIFU transducer, 6. Acoustic gel cushion for acoustic coupling, 7. Degassed water for acoustic coupling, 8. MR loop coil, 9. Basal support for coil and reservoir. (d) Illustration of a low power HIFU sonication in the transverse plane as seen by PRFS MR thermometry. The white arrow indicates the planar interface between the acoustic gel cushion (6) and the gel matrix of the perfused model, visualizing minimal heating. The dotted red line delimitates the cross section of artificial capillaries region.

The model was bi-compartmental, consisting of a quasi-periodic anisotropic structure made from semi-rigid gel and a fluid circulation through the interstices. The semi-rigid gel was prepared from degassed water, 11.2% w/w of glycerol (Acros Organics, Thermo Fischer Scientific, Geel, Belgium), 0.05% w/w of benzalkonium chloride (BAL) (Sigma-Aldrich, St. Quentin Fallavier, France) and 3% w/w of agar (Alfa Aesar, Karlsruhe, Germany). The BAL, glycerol and degassed water were added to a 400 ml tared beaker. Agar was then added and the mixture was mechanically stirred and heated at 90 °C for 1 h. The agar-based matrix provides rigidity and impermeability to sonosensitizers. Furthermore, 15% w/w of evaporated milk (8.5% fat, 7.1% proteins) was added to the preparation to provide stable acoustic behavior, e.g., controlled acoustic attenuation, according to Menikou et al. [59]. The fluid mixture was maintained at approximately 80 °C and was aspirated inside stainless steel biopsy sheath of 1.7 mm inner diameter × 70 mm, then rapidly cooled in ice bath to form strands of tissue-mimicking gel. Strands were extracted from the sheath and densely packed approaching a hexagonal symmetry (corresponding to 90% of tissue-mimicking gel and 10% of interstitial gap) inside a cylindrical cavity of 20 mm inner diameter. The cavity was created inside a 1.5% w/w agar gel matrix which filled a 3 D-printed holder, and was designed to avoid motion during HIFU sonications and vibrations from MR gradients. This geometry mimicked the tissue structure irrigated by micro-circulation in capillaries (Figures 2 and 3). The model was perfused with an MR-compatible perfusion machine (Figure 4) according to Buchs et al. [60]. The main difficulty of our model was the presence of trapped air bubbles, which create a barrier for HIFU and susceptibility artifacts on MR images. To resolve this, the gel matrix was prepared with degassed water, the *in vitro* model of perfused tissue was further degassed at least 30 min by connecting to a vacuum-circulating pump, and the cylindrical cavity hosting the strands was orientated slightly inclined down-up, to facilitate evacuation of air bubbles at the upper edge. A 3D gradient echo high resolution MRI was used to confirm the absence of interstitial bubbles before any sonication being performed (Figure 3).

### 2.3. Perfusion of tissue models

Appropriate ethical approval was obtained for animal sacrifice (adult pig, *n* = 4) and organ excision and preservation on ice bath.

The MR-compatible perfusion machine (Figure 4(a)), comprised a drive module with automatic feedback control of pressure, an ‘umbilical cord’ connecting the drive module and a perfusion module as described by Buchs et al. [60]. We connected the perfusion machine to the described tissue-mimicking model, to freshly excised pig kidneys (*n* = 3) and freshly excised pig liver (*n* = 1). Each MRgHIFU experiment was performed at room temperature.

The flow rate was adjustable in the range of 0–1 ml/s. During sonication stages, this value was set to 0, 0.1 or 0.15 ml/s, corresponding to 0, 3 or 4.5 mm/s for the *in vitro* model. These values are significantly lower, but still comparable with the human renal medulla perfusion rate [61]. The perfusate velocity cannot exceed some experimental limitations, in order to enable appropriate heat exchange with the semi-rigid gel strands that are observed by PRFS thermometry, as explained in the Discussion. The purpose of the perfusate circulation was the sonosensitizers delivery in the interstitial spaces rather than providing an effective tissue cooling. Therefore, this flow rate difference does not affect the conclusions with respect to the activity of sonosensitizers.

**Figure 5.**
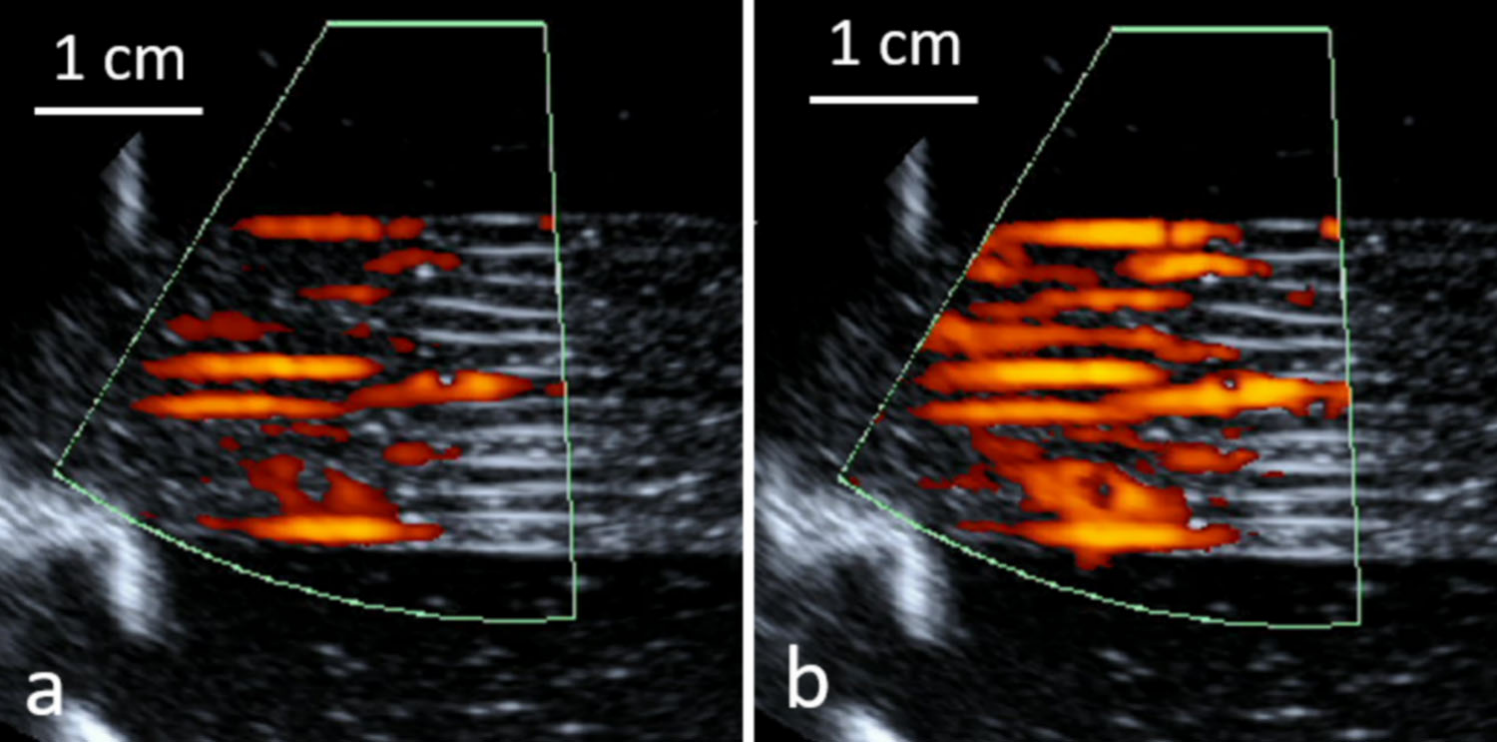
Laminar flow of the in vitro model of perfused tissue observed by pulsed color Doppler ultrasonography during a phase of ‘diastole’ (a) and ‘systole’ (b). Carrier frequency 5 MHz, pulse repetition frequency 100 Hz.

During the degassing stage of the tissue-mimicking model, the flow rate was set to maximum. The laminar flow in the tissue-mimicking model was confirmed by pulsed-color echo Doppler as shown in Figure 5, as well as by phased contrast MR flow imaging (data not shown).

In the renal perfusion setup, the flow input was the renal artery while the output was the renal vein. In the hepatic perfusion setup, the hepatic artery was clamped, the flow input was the portal vein while the output were the three branches of the sub-hepatic vein collected in a segment of the inferior vena cava.

One or two doses of sonosensitizers were injected in the perfusate volume (V) resulting in v/v concentrations c1 and c2 as follows: V(mL)/c1(%)/c2(%) = 250/0.1/0.2 in the tissue-mimicking model, 750/0.125/0.235 in excised kidneys and 750/0.1/0.188 in excised liver.

The perfusion pressure was recorded after each injection of sonosensitizers in *ex vivo* organs to assess the hydrodynamic resistance and thus an effective intra-capillary circulation. The intra-renal and intra-hepatic microcirculation was assessed at the end of the experiment by injection of gadolinium chelate based on the report by Buchs et al. [62].

### 2.4. Focused ultrasound

Focused ultrasound was generated by an MR-compatible 256-element pseudo-randomized phased array transducer (Imasonic, Besançon, France) of radius 130 mm and diameter 140 mm. The frequency of ultrasound was 1031 kHz and the transducer was powered by a 256-channel beam former (Image Guided Therapy, Pessac, France). Two modes of ablation were defined with different acoustic parameters to deliver the energy in the perfused tissue mimicking model and in *ex vivo* organs. In the first condition, the HIFU beam targeted a fixed focal point. In the second condition, the energy was delivered in a circular pattern defined by a 4 mm-diameter circle described by 16 equidistant points. The parameters used for HIFU treatment ranged from 10 to 33 s for the total duration and from 60 to 135 W acoustic power, with a duty cycle between 70% and 94%. Based on Saletes et al. [63], the acoustic intensity ranged from 432 W cm^−2^ to 972 W cm^−2^ with negative peak pressure between 3.2 MPa and 4.6 MPa, and positive peak pressure between 4.1 MPa and 6.8 MPa.

The tissue-mimicking model was sonicated by targeting the central axis of the cylindrical pack of strands as shown in Figure 3(a,b). The matrix gel surrounding the gel strands was sufficiently large to avoid boundary conditions.

The perfusion machine was adapted for acoustic coupling to HIFU and a dedicated underlying support was designed to maintain the MR receive loop coil close to the organ (Figure 4). The perfused models were topped with acoustic gel to avoid beam reflections at interfaces [64]. As shown in Figure 4(b,c), the acoustic coupling between the HIFU and the perfused tissue model was performed by placing an acoustic gel cushion between the HIFU membrane and the perfusion machine.

In *ex vivo* experiments the HIFU beam independently targeted the proximal cortex of the kidney, the hepatic parenchyma or the middle hepatic vein.

A total of 44 sonications were performed and analyzed in the tissue-mimicking model, 49 in the cortex of excised kidneys, 15 in the excised liver parenchyma and 7 in the excised liver middle hepatic vein.

### 2.5. MR imaging

Data were acquired using a 3 T whole body MR scanner (Prisma Fit, Siemens, Erlangen, Germany) equipped with an 11 cm-diameter single element loop coil.

**Figure 6.**
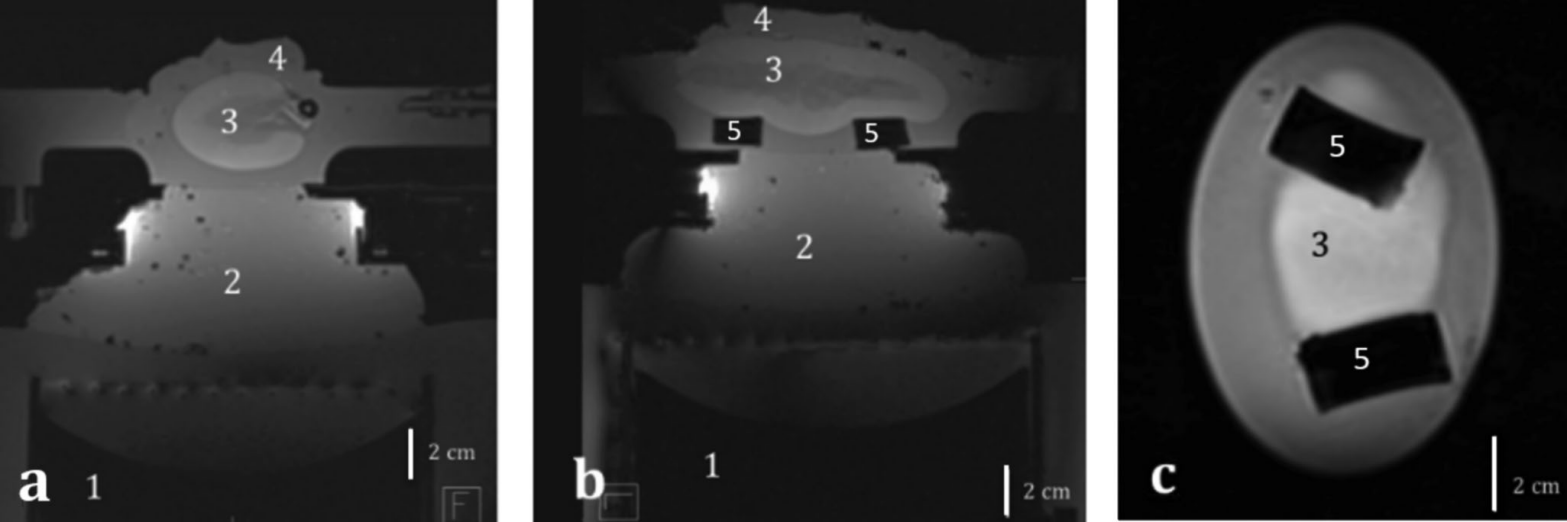
T1 MR images of an excised ex vivo perfused kidney in (a) transversal, (b) sagittal and (c) coronal plane. 1. HIFU transducer, 2. Standard ultrasonic gel for acoustic coupling, 3. Perfused organ, 4. Standard ultrasonic gel for acoustic wave dissipation, 5. Soft supports for the organ.

High-resolution 3D T1-weighted images (T1-VIBE) were acquired for the positioning and targeting of the focal point and control of the sample degassing (Figure 3 for the *in vitro* model and Figure 6 for the *ex vivo* model). Main parameters were as follows: repetition time (TR) = 5.44 ms, echo time (TE) =2.46 ms, flip angle = 10°, spatial resolution = 0.86 mm isotropic, TA = 250 s, BW = 390 Hz/pixel, PF phase/slice = 7/8.

A high-resolution 2D turbo spin echo T2-weighted sequence (T2-TSE) was acquired perpendicular to the strands (transverse orientation) for accurate assessment of their packing. Main parameters were as follows: TR = 5000 ms, TE = 117 ms, slice thickness = 1 mm, number of slices = 12, refocusing angle = 146°, acquired resolution = 0.5 × 0.5 mm^2^, interpolated to 0.25 × 0.25 mm^2^, TA = 387 s, NSA = 4, BW = 160 Hz/pixel, turbo factor = 17.

MR thermometry was performed using the PRFS method, based on temperature dependent phase changes [8]. Main parameters were as follows: TE = 8.62 ms, TR = 41.38 ms, number of averages = 1, slice thickness = 5 mm, flip angle = 20°, FOV = 128 × 128, in plane spatial resolution = 1 × 1mm^2^, FATSAT lipid signal suppression, bandwidth = 814 Hz/Px. Magnitude and phase images were sent in real-time to a PC running Thermoguide software (Image Guided Therapy, Pessac, France) and merged with temperature maps to monitor temperature changes in the tissue. Pilot sonications were monitored in both axial and coronal planes to confirm the targeting, the regular shape of the focal spot and the precision of temperature monitoring (Figure 4(d)).

The assessment of perfusion was evaluated at the end of the experiment by using dynamic contrast enhanced (DCE) MRI *via* injection of 2 ml of 0.5 mmol gadolinium chelate bolus (Dotarem, Guerbet, France) with the perfusate. The parameters of the dynamic 2D saturation-prepared turbo flash sequence for the kidney were: inversion time = 240 ms, flip angle = 12°, spatial resolution = 1.0 × 1.3 mm^2^, temporal resolution = 3.45 s, number of slices = 5, slice thickness = 4 mm, TR = 460 ms, TE = 1.3 ms, number of dynamics = 140 for a total duration of 5 min 48 s; and for the liver were: inversion time = 255 ms, flip angle = 12°, spatial resolution = 1.25 × 1.25 mm^2^, temporal resolution = 3.0 s, number of slices = 6, slice thickness = 4 mm, TR = 501 ms, TE = 1.44 ms, number of dynamics = 140 for a total duration of 5 min 48 s.

### 2.6. Post-processing

The effect of the enhancement of the HIFU-induced temperature elevation after injection of sonosensitizers was evaluated by defining two metrics similar to [57]. The first metric (units: °C/kJ) corresponds to the ratio between the temperature elevation at the focal point when sonication ended (${\Delta T}_{\max},$ units °C), and the emitted acoustic energy (E,  units kJ):

[1] $$\mathrm{Metric}\#1=\frac{{\Delta T}_{\max}}{E\;}$$

The focal point temperature elevation was de-noised pixel-wise by using a moving average filter with span of 5. Metric#1 estimates the improvement of capability to induce an ablation at the direct target.

The second metric (dimensionless) represents the fraction of thermal energy deposited at the end-point of HIFU sonication in the MR slice relative to the emitted acoustic energy:

[2] $$\mathrm{Metric}\#2=\frac{\sum_{1}^{N}{\Delta T}_{i}}{E}*c_{m}*\;\rho *V_{P}$$

where N = number of voxels, $\Delta T_{i}=\;$ temperature elevation (°C) in the voxel *i* corresponding to the end-point of the HIFU sonication, if a voxel-wise threshold of 0.5 °C is exceeded; $c_{m}$ = specific heat capacity (kJ. kg^−1^. °C^−1^), ρ = average density of the voxel (kg.*m*^−3^), $V_{P}$ = volume of one voxel (m^3^). Metric#2 is a global estimation of the energy deposition, considering that through-plane heat diffusion has a specific time of several minutes and can be neglected during the time of active sonication, a few tens of seconds. This hypothesis is justified by the elongated shape of the focal spot [65]. Metrics#2 is invariant in first approximation against in-plane heat flow (e.g., fluid flow), given the energy conservation principle.

The definition of these metrics allows the standardization of the HIFU energy deposition. To evaluate the effect of the injection of sonosensitizers, we compared the resulting metrics between scenarios without and with sonosensitizer injection at different concentrations using Student’s *t*-test for independent samples. Statistical tests were performed with the SPSS Statistics 24.0 software (IBM, Armonk, New York, USA). A two-tailed *p* value <0.05 was considered as significant.

Data of DCE imaging were analyzed with OsiriX software (v3.8.1, Bernex, Switzerland) by plotting Gd-uptake curves to assess the efficiency of the perfusion in excised organs according to Buchs et al. [62]. In the liver, regions of interest (ROI) were drawn on the portal vein branches and the hepatic parenchyma. In the kidneys, ROI were drawn on the cortex and the medulla. For the medulla, the ROI must avoid the urinary excretory system and the hilum (internal limit). After plotting the post-injection signal enhancement curves, two features were evaluated to conclude on the viability of the kidneys [62]. These were the negative slope of perfusion in the cortex (in negative trigonometric degrees) and the time of arrival of the contrast agent bolus in the cortex, which should be shorter than the same parameter in the medulla.

## 3. Results

### 3.1. Sonosensitizers

Mean droplet size ranged from 1.42 to 2.16 µm diameter in different batches, with intra batch diameter standard deviation of approximately 0.63 µm. An example size distribution histogram is shown in Figure 1. The size distribution was found to be stable for at least 30 days of storage between 2 and 4 °C in a concentrated emulsion of 2% v/v. Based on the time duration to conduct the experiment, diluted emulsions of 0.1 to 0.2% v/v of sonosensitizers are stable at least 90 min at 25 °C.

### 3.2. Tissue-mimicking model

T1-VIBE images acquired after degassing the *in vitro* model showed the suppression of macroscopic air bubbles in the interstitial compartment (Figure 3(a–c)). The high resolution T2-TSE (Figure 3(d)) allowed visualization of the regular packing of strands and evaluation of the volume filling ratio gel:fluid at approximately 9:1. A perfectly hexagonal 2D lattice would yield gel:fluid ratio of 10:1.

### 3.3. Enhancement of HIFU thermal effect

Our results show an improvement in the thermal deposition after injection of sonosensitizers for each condition. The shape of the focal spot was not specifically distorted by the administration of sonosensitizers.

**Figure 7.**
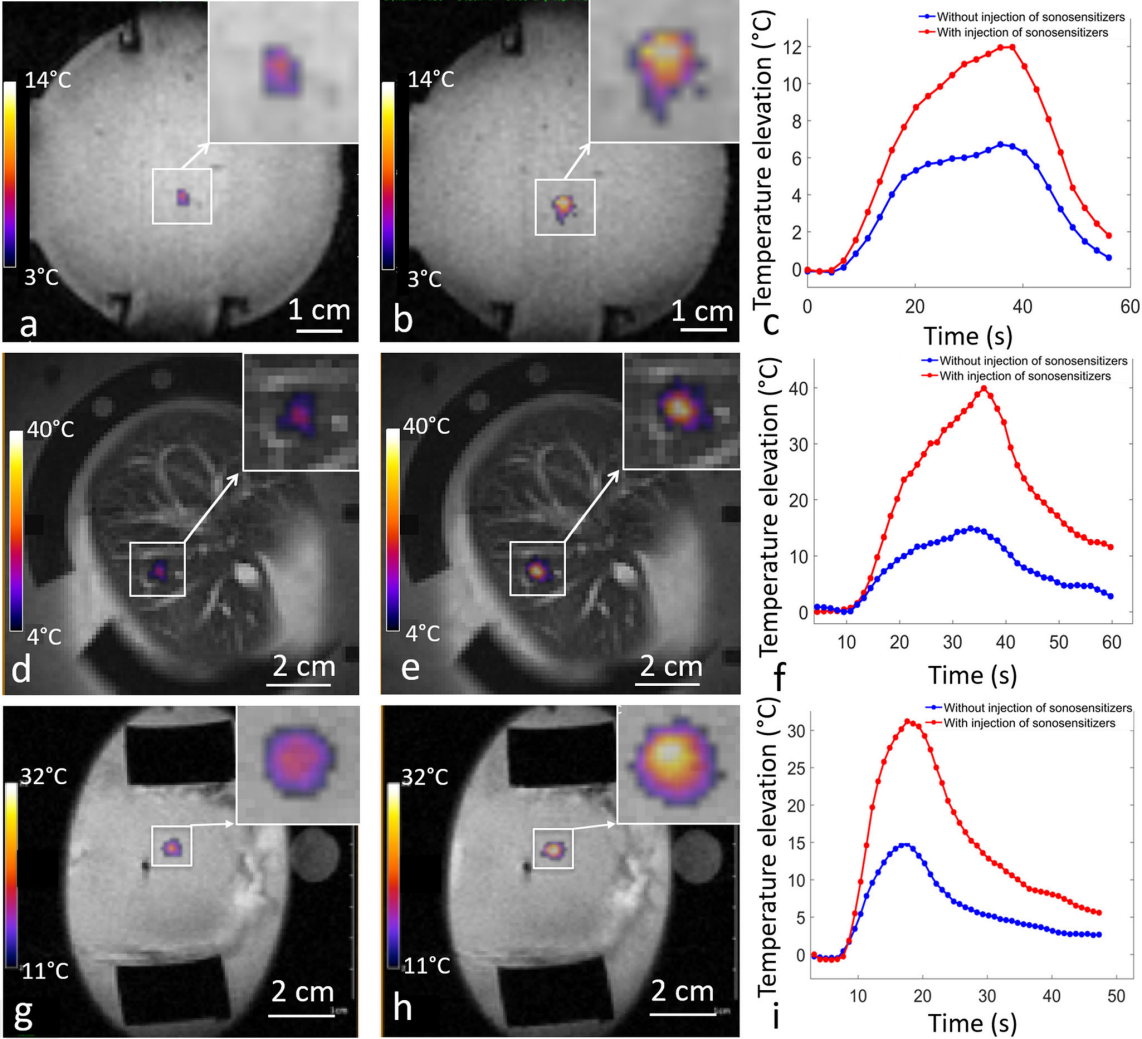
Temperature maps at the end-point in the perfused tissues, (a,d,g) without injection and (b,e,h) with injection of sonosensitizers. (c,f,i) temperature elevation as function of the time without injection (blue line) and after injection of one dose of sonosensitizers (red line), in the in vitro model of perfused tissue (1st row. 0 vs. 0.1%, fixed focus, 60 W), in the liver (2nd row. 0 vs. 0.188%, fixed focus, 94 W) and kidney (3rd row. 0. vs 0.235%, fixed focus, 135 W).

In the *in vitro* model of perfused tissue (Figure 7(a–c) and Table 1 for detailed results and statistics), the differential metric#1 due to the injection of 0.1% of sonosensitizers was 2.29 °C/kJ on average, for fixed focal point sonication. The differential metric#2 was 3.28 J/kJ. Injection of 0.2% of sonosensitizers induced a differential metric#1 of 2.63 °C/kJ and differential metric#2 of 5.24 J/kJ. These results demonstrated a significant enhancement in the thermal deposition after injection of sonosensitizers (*p* < 0.00001). Repeated time-delayed sonications indicated that the pool of circulating droplets was stable for at least one hour following administration, in the range of concentration 0 to 0.2% v/v.

**Table 1.** Overview of the differential effect of sonosensitizers on metric#1 and #2 for the *in vitro* model of perfused tissue. The average value and the corresponding confidence interval (CI) for a given number of sonications are provided.

Batch	Sonication pattern	Perfusion rate (mL/s)	Concentration of sonosensitizers (% v/v)	Number of sonications	Increase of Metric#1 and CI 10–90 (°C/kJ)	Increase of Metric#2 and CI 10–90 (J/kJ)	Increase of Metric#2 Normalized to Concentration (mJ/kJ/ppm)
1	Fixed focal point	0.1	0.1	6	2.49	2.94	294
					[2.12–2.86]	[2.55–3.33]	
2	Fixed focal point	0.15	0.1	5	2.09	3.63	363
					[1.66–2.52]	[2.81–4.45]	
2	Fixed focal point	0.15	0.2	4	2.63	5.24	262
					[1.81–3.45]	[4.38–6.11]	
2	Circular 16 foci	0.0	0.1	5	1.30	2.49	249
					[0.62–1.99]	[0.74–4.25]	
2	Circular 16 foci	0.1	0.1	6	0.59	2.33	233
					[0.39–0.78]	[1.95–2.70]	

In *ex vivo* perfused kidneys (Figure 7(g–i) and Table 2), the differential metric#1 due to the injection of 0.125% of sonosensitizers was found 14.5 °C/kJ on average for the fixed focal point and 2.5 °C/kJ on average for the circular pattern. The corresponding differential metric#2 was 3.5 J/kJ and 1.8 J/kJ for the fixed focal point and for the circular pattern respectively. The differential metric#1 due to the injection of 0.235% of sonosensitizers was 20.0 °C/kJ on average for the fixed focal point and 7.8 °C/kJ on average for the circular pattern. The corresponding differential metric#2 was 6.0 J/kJ and 4.9 J/kJ for the fixed focal point and for the circular pattern, respectively. Overall, the administration of 0.235% sonosensitizers multiplied by a factor of 3 the ultrasound absorption in the renal cortex.

**Table 2.** Overview of metric#1 and #2 in 3 specimens of *ex vivo* perfused kidney, series of independent sonications, same condition per line.

Sonication pattern	Concentration of sonosensitizers (% v/v)	Number of sonications	Metric#1 and SD (°C/kJ)	Metric#2 and SD (J/kJ)	Increase of Metric#2 Normalized to Concentration (mJ/kJ/ppm)
Fixed focal point	0	10	9.8 ± 3.7	3.0 ± 0.8	–
	0.125	11	24.3 ± 13.1	6.5 ± 1.0	280
	0.235	9	29.8 ± 15.7	9.0 ± 3.8	255
Circular 16 foci	0	6	3.5 ± 0.5	3.4 ± 0.2	–
	0.125	3	6.0 ± 2.1	5.2 ± 1.1	144
	0.235	10	11.3 ± 2.9	8.3 ± 2.6	208

In the *ex vivo* liver (Figure 7(d–f) and Table 3), the differential metric#1 for the fixed focal point sonication due to the injection of sonosensitizers was found 1.0 °C/kJ at 0.1% v/v and 10.3 °C/kJ at 0.188% v/v. The differential metric#2 was 1.0 J/kJ at 0.1% v/v and 3.9 J/kJ at 0.188% v/v. The differential metric#1 for the circular pattern of sonication due to the injection of 0.188% sonosensitizers was 3.0 °C/kJ while the differential metric#2 was 1.1 J/kJ.

**Table 3.** Overview of metric#1 and #2 calculated in one *ex vivo* perfused liver specimen, series of independent sonications.

Sonication pattern	Concentration of sonosensitizers (% v/v)	Metric#1 per sonication (°C/kJ)	Metric#2 per sonication (J/kJ)	Increase of Metric#2 Normalized to Concentration (mJ/kJ/ppm)
Fixed focal point	0	6.6	3.6	
		6.9	2.7	–
	0.1	8.5	4.6	
		7.3	4.4	107
		7.7	3.5	
	0.188	16.1	8.3	
		16.1	6.4	211
		17.4	6.6	
		18.8	7.0	
Circular 16 foci	0	7.8	7.7	–
		7.0	8.3	
	0.188	11.6	11.7	
		10.0	8.1	58
		10.0	8.1	
		10.2	8.5	

When the HIFU beam targeted a fixed focal point in a middle hepatic vein, an improvement of metric#1 was not observed while the differential metric#2 was on average 0.6 J/kJ for the double dose of sonosensitizers, indicating minor enhancement of heating effects as shown in Table 4.

**Table 4.** Metric#1 and #2 measured for a fixed focal point sonication targeting a middle hepatic vein.

Concentration of sonosensitizers (% v/v)	Metric#1 (°C/kJ)	Metric#2 (J/kJ)
0	4.7	2.4
	5.8	4.1
	3.3	3.5
0.188	6.0	4.5
	4.3	4.1
	4.1	5.8
	3.8	6.3

**Figure 8.**
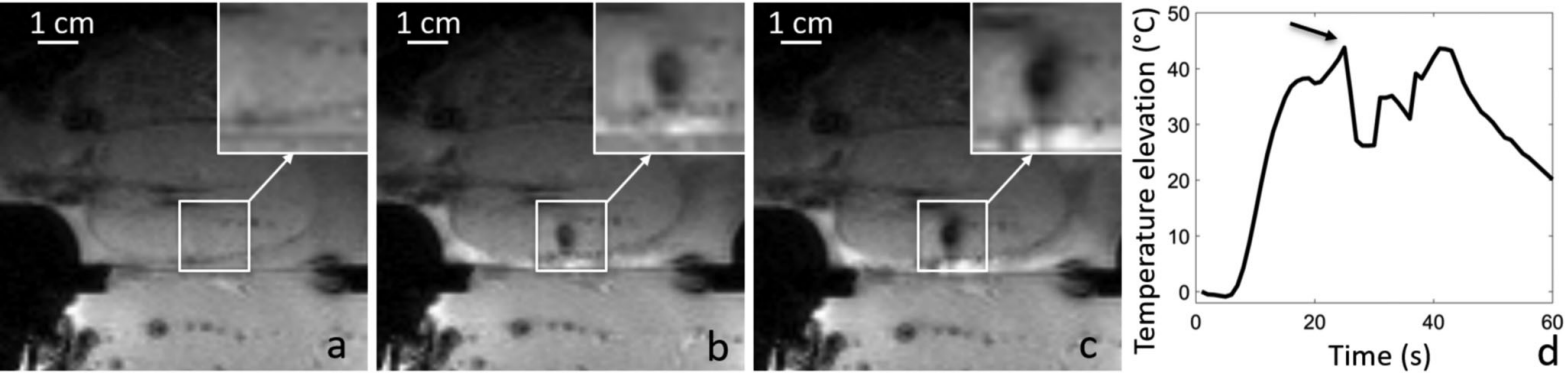
Illustration of a boiling core produced after injection of sonosensitizers in a kidney. Magnitude MR GRE data is shown (a) before the HIFU sonication, (b) 7s after the beginning of the 30s HIFU sonication. The observed MR phase at focal point is disrupted after the onset of the boiling core, as shown with the black arrow in plot d).

For HIFU sonication in the excised kidney with 60 W or 135 W steady acoustic power, after injection of a double dose of sonosensitizers (0.235% v/v in the perfusate), a macroscopic ‘boiling core’ within the cortical tissue was created, visible as hyposignal on magnitude images, as illustrated in Figure 8 and Supplementary file. This phenomenon was observed in 4 sonications, different locations, same excised kidney. Table 5 details the sonication time to onset (range: 8–22 s) and the highest temperature elevation still measurable by PRFS before the disruption of MR signal (range: 29–51 °C). The respective temperature elevation is to be compared with less than 10 °C without sonosensitizers, that is, a multiplicative factor ranging from 3 to 5. After the occurrence of the boiling core, water evaporation and emergence of a vapor bubble cloud in the tissue induced susceptibility artifact, observed on T2*-weighted images, identifiable by signal loss or void, as well as discontinuities and unreal fluctuations of the complex signal phase. Occurrence of a macroscopic ‘boiling core’ correlates with a high temperature reached locally, due to preferential energy deposition in the inferior part of the focal area from the forward wave and reflected wave at the bubble cloud interface. A similar effect was reported by Viallon et al. [64] for a non-perfused liver tissue using a higher acoustic power at the same frequency (200 W average).

**Table 5.** Elapsed time from the beginning of steady power sonication and temperature elevation at the time point of occurrence of a macroscopic ‘boiling core’, in a perfused kidney using 0.235% v/v sonosensitizers.

Sonication ID	Acoustic power [W]	Elapsed time (s)	Temperature elevation (°C)
1	135	17.1	43.8
2	135	8.1	28.9
3	135	21.6	31.6
4	60	18.9	50.9

### 3.4. Perfusion of *ex vivo* organs

Systolic pressure (40 mmHg) and diastolic pressure (20 mmHg) in the perfusion system remained unchanged after injection of one or two doses of sonosensitizers in excised organs. This result indicates that no overload of the stabilized flow rate occurred and, thus, no observable obstruction of capillaries, if any. Note, however, these pressure values were significantly lower than the human physiological values to avoid the glomerular filtration and/or damage.

**Figure 9.**
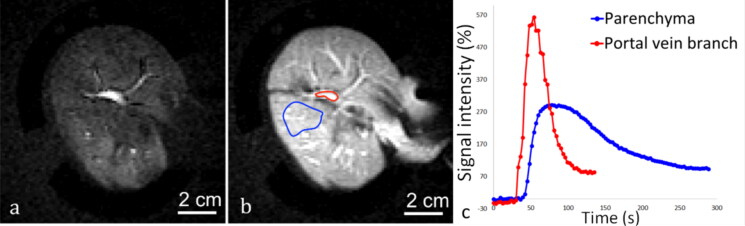
2D saturation-prepared turbo flash sequence after gadolinium chelate injection applied to the liver, after the organ was perfused with 0.188% sonosensitizers. DCE data is shown (a) 15 s after bolus injection, (b) 30 s after bolus injection. c) Signal enhancement curves represent the signal intensity in a ROI on a portal vein branch (red line) and the hepatic parenchyma (blue line).

**Figure 10.**
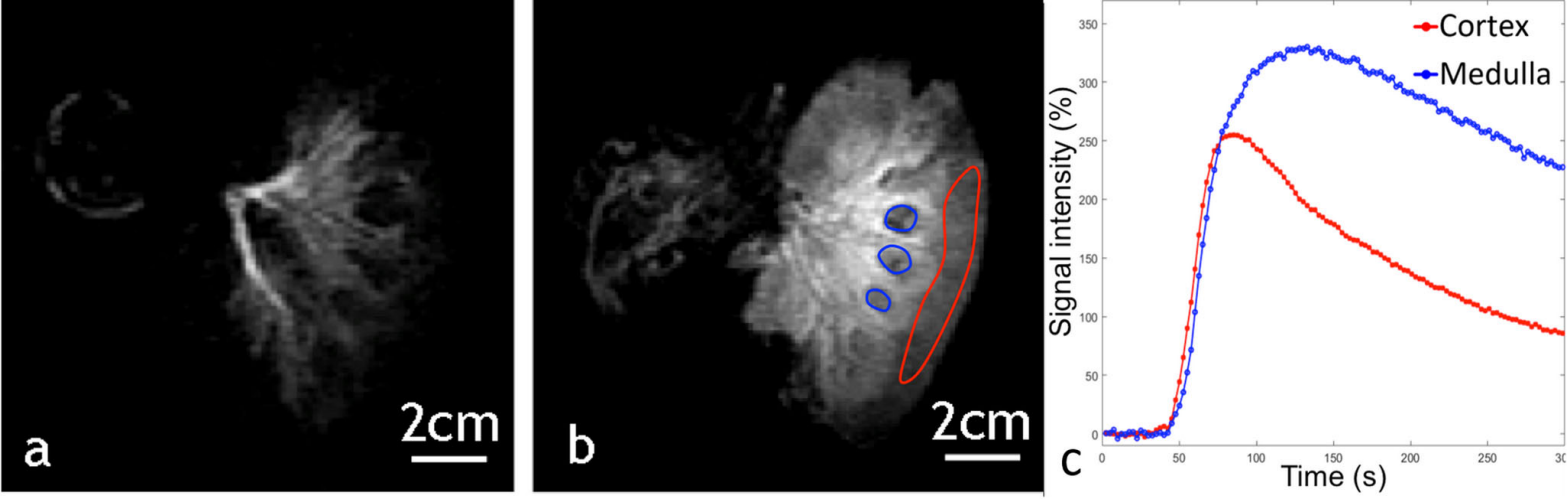
Example of a 2 D saturation-prepared turbo flash sequence after gadolinium chelate injection applied to ex vivo kidney, perfused with 0.235% sonosensitizers at (a) 15 s after bolus injection, (b) 1 min after bolus injection. (c) Signal enhancement curves in an ROI set in the medulla (blue) and the renal cortex (red).

Gadolinium uptake DCE images demonstrated an effective circulation of sonosensitizers in the capillary network of excised organs and absence of ischemia as shown in Figures 9 and 10. Signal enhancement curves of each kidney indicated that in all cases the bolus time of arrival is shorter in the cortex than in the medulla, and the angle between the maximum signal value in the cortex and at the end of flushing were −51°, −62° and 29° in the three kidney specimens, according to Buchs et al. analysis [62].

## 4. Discussion

### 4.1. Enhancement of HIFU thermal effect by liquid-core micron-sized droplets

This study confirmed the capabilities of PFOB-FTAC micron size droplets to enhance the local absorption of focused ultrasound, as already reported by Desgranges [57]. Differential metrics with and without injection of sonosensitizers showed a significant improvement in the temperature elevation achieved, inducing a dramatic amplification of the thermal dose in the target area. As the thermal dose scales exponentially with temperature, a factor of 2 per degree Celsius [66], an additional peak temperature increase of 15 to 25 °C, as shown in Figure 7(f,i), means at least three orders of magnitude higher thermal dose.

When comparing quantitatively the metrics of absorption enhancement divided by the effective volume ratio of this new formulation of sonosensitizers, we report approximately 6-fold higher efficiency than the initial formulation of Desgranges et al. [57]. Indeed, the volume fraction of sonosensitizers here is provided relative to the circulating fluid compartment, and this fluid only fills approximately 10% of the perfused tissue-mimicking model. In the previous report, the volume fraction of sonosensitizers was provided relative to the entire bulk volume of the doped gel.

The ratio of metric#2 enhancement to the micro-droplet volume concentration in perfusate, according to Tables 1–3, indicates a good correlation of the acoustic sonosensitizers activity between the *in vitro* and *ex vivo* models.

Further increasing the volume fraction of sonosensitizers above 0.2% in the perfusion fluid in the tissue-mimicking model did not yield additional benefit for the HIFU thermal therapy (data not shown). Quantitative results illustrating this tendency of saturation of the effect are outside the scope of this report. Due to practical reasons and aiming for future clinical feasibility, the concentration of sonosensitizers should remain as low as possible even if the resulting effect would linearly increase.

Injection of a fraction of sonosensitizers of 0.125% in the perfused *ex vivo* kidney with fixed focus sonication yielded in average 2.5 times higher temperature elevation at the focal point than without sonosensitizers, according to Table 2, second raw vs. first raw. The fluid volume fraction at the specific location of sonication was not accurately known; however, considering an estimate of 10% blood volume in renal cortex [67] this means an estimated 0.0125% v/v sonosensitizers versus bulk tissue and, therefore, an individual effect of acoustic absorption by inserting one micro-droplet, under high dilution conditions, at least 10’000 times higher than the equivalent volume of tissue itself.

The physical mechanism of enhanced energy deposition in presence of our sonosensitizers was not investigated in this study. Correlating the observation from Desgranges [57] on the steady micro-droplet response -to repeated HIFU sonication and the observations from the current study on the saturation effect above 0.2% v/v concentration in our ‘artificial kidney’, provides insight to the physical mechanism. We hypothesize physical phenomena yielding enhanced absorption in the external space adjacent to the micro-droplet and sonosensitizer acoustic interaction cross section at least one order of magnitude larger than the geometrical section. Facilitated cavitation, streaming and shear waves could be involved in varying proportions. Better understanding of these phenomena would further enable the improvement of the design of this class of sonosensitizers, aiming at higher efficiency and thus a lower administered dose.

The minimal effect of heating enhancement when the focal point targeted a middle hepatic vein suggests that the competition between the average local concentration of sonosensitizers and the heat sink of the fluid flow is not favorable for large vessels. Therefore, perivascular tumoral tissue lying in the immediate vicinity of large vessels may not be an appropriate target for the current approach.

These results are encouraging for future translation to *in vivo*, although the currently administrated concentration of sonosensitizers in the ‘blood’ of the order of 1:1000 v/v may be too high. On the other hand, multiplying the temperature elevation by a factor of 2.5 is not required in a clinical application because the thermal dose scales exponentially with temperature, so a 50% additional temperature elevation (that is a factor 1.5) would be a significant result. The useful concentration of sonosensitizers would therefore scale down to an estimate of 1:3000 v/v. This volume fraction would be achieved in an adult patient of 70 kg by an i.v. injection of 90 ml of PFOB-TAC emulsion concentrated at 2% v/v. The blood volume change would be minimal. The corresponding doses of PFOB and FTAC would be far below the extrapolated LD50 from rats, by a factor of several hundred and several thousand, respectively [68,69].

### 4.2. Role of continuous flow of fluid in the ‘vascular’ compartment

Fluid circulation in the organs is essential to provide a continuous supply of sonosensitizers at the focal spot, leading to a steady response a few minutes after iv injection. In return, the endovascular sonosensitizers must not produce occlusion of capillary network. Our DCE MRI data demonstrated a continuous fluid flow in excised and perfused pig kidney after injection of 0.235% v/v sonosensitizers and, in pig liver, 0.188% v/v.

The additional heat generation from the interaction of endovascular sonosensitizers with the HIFU beam occurs inside the ‘vascular’ compartment, while the vast majority of water protons observed by PRFS MR thermometry does not belong to that compartment. The ratio of endovascular versus total number of observed protons was estimated at 1:10 for the tissue-mimicking model and kidney cortex. Therefore, the macroscopic PRFS MR thermometry may underestimate the actual additional heat, because a heated fluid may leave the region of interest without reaching a thermal equilibrium with the bulk tissue. This is of particular concern in the tissue-mimicking model, where the flow is laminar and the diameter of the ‘capillaries’ is half millimeter, at least one order of magnitude larger than biological capillaries. The size of ‘capillaries’ can be estimated theoretically for a hexagonal 2d lattice and visualized in Figure 3(d), that is, the T2 hyperintense signal between the gel strands. In other words, the characteristic time of thermal coupling between interstitial fluid and gel strands has to be discussed, as well as the average displacement of the circulating fluid during the characteristic time of thermal equilibrium. Using the Green function's formalism [65], the heat diffusion thermal equilibrium for a half-millimeter diameter ‘capillary’ is estimated at 0.25 s. Considering the flow rate of 0.1 ml/s and the free section of flow between gel strands of 0.33 cm^2^ according to the geometry of the model, the average flow velocity is 3 mm/s. Subsequently, the average displacement of fluid (and transported sonosensitizers) until the heat is transferred to the bulk tissue is less than 1 mm. This distance is totally encompassed in the ROI where the spatial integral of temperature elevation is calculated for metric#2, still this is a 2 D in-plane ROI. Theoretically, the PRFS MR thermometry-based metrics, as defined previously, should be representative for the additional heat generation and only a very small fraction of thermal energy may ‘escape’ to this quantification *via* a through-plane slow diffusion mechanism. Experimentally, this situation was confirmed by the data shown in Table 1, as the differential metric#2 due to injection of 0.1% v/v sonosensitizers with and, without flow was very similar, 2.33±(SD)0.27 J/kJ and respectively 2.49±(SD)0.75 J/kJ. The two-tailed alternative hypothesis was rejected by an independent sample T-test. Figure 7(c) (zoomed inset) shows a minor distortion ‘coma-like’ of the focal spot, that appeared elongated in the flow direction instead of a circular symmetry. This minor distortion is not impacting the calculation of metric#2, which integrates the global temperature elevation in the slice.

### 4.3. Reproducibility

Repeated sonications at the same location in perfused *in vitro* model showed reproducible heating effect within 10% for a given configuration and within 20% for the differential metrics (Table 1), that was largely sufficient to demonstrate the relative effect of sonosensitizers. One should note that the physical interaction between the HIFU beam and the sonosensitizers occurs in the compartment of circulating fluid and should not trigger disruptive effects on the gel strands, as far as the local temperature does not approach the melting point of that gel.

The ratio of differential metric#2 to the volume concentration of sonosensitizers is coherent between the *in vitro* model of perfused tissue and *ex vivo* perfused kidney, averaging 0.0328 J/kJ/ppm *in vitro* and 0.028 J/kJ/ppm in *ex vivo* kidney, fixed focal point sonication.

The evaluation of MR contrast agent time of arrival and the negative slope calculation on DCE perfusion curves indicate that vascular function was variable between *ex vivo* samples but sufficient in every case for the purpose of the current study, that is for the continuous supply of sonosensitizers

### 4.4. Relevance of the model

The tissue-mimicking perfused gel is considered an appropriate model for the HIFU interaction with perfused biologic tissue, potentially sparing some *in vivo* experiments, with the advantages of being re-usable, offering stable properties and having a tunable perfusion rate. Moreover, this model of perfused organ may be used for studying different types and variants of sonosensitizers, as well as their acoustic behavior as a function of v/v concentration, proportion of dissolved gases, proportion of macro-molecules in the perfusate etc.

### 4.5. Limitations of the study

The concentration of the circulating sonosensitizers has not been measured *in situ* inside the perfused organs in this study. Fluorescent markers or ^19^F MRI of the PFOB signal [69] may be used for this purpose. Instead, we considered that the respective concentration is the same inside and outside the organs, according to the physical dilution of calibrated emulsions.

The average diameter in different batches of sonosensitizers varied within ± 20%, suggesting that the intrinsic acoustic behavior was not strictly uniform among experiments. For future *in vivo* studies, we are suggesting batch-specific pre-testing on the tissue-mimicking phantom, before the animal experiment, to assess the actual efficacy of enhancing the HIFU absorption.

In excised organs, the spatial inhomogeneity of tissue played a potential role in increasing the standard deviation of results shown in Tables 2 and 3. It is acknowledged that the local fluid fraction and flow rate are variable. Overall, it is important to note that the enhancing effect of sonosensitizers largely exceeded the experimental variability.

As the chemical composition of the perfusate used in this study is different from blood, the behavior of sonosensitizers may be modified *in vivo* and specific translational studies are therefore needed.

## 5. Conclusion

Temperature elevation maps clearly demonstrated a dramatic increase in the achieved temperature after administration of described sonosensitizers in experimental models *in vitro* and *ex vivo*. Under specific conditions, the enhanced HIFU thermal effect yielded water boiling in tissue. The quantitative metrics of the enhanced thermal effect together with the long circulating time of sonosensitizers in tissue mimicking models suggest a future interest for *in vivo* studies. However, the stability of these sonosensitizers and their long *in vivo* circulation time still needs to be established. Reaching higher temperature with lower HIFU power may help to simplify technological constraints and reduce the risks for near-/far-field heating. Tissue perfusion is traditionally considered a challenge for the thermal therapy; however, the administration of endovascular sonosensitizers that are stable in the blood pool during the therapy may turn it into an advantage. We also report an experimental model that is suitable for other MR-guided HIFU studies of new methods and applications.

## Supplementary Material

Boiling Core Animation

## Data Availability

The datasets supporting the conclusions of this article are available in https://yareta.unige.ch/frontend/archive/b76ea208-be70-427e-8a67-281774e04e07
