# Supplementary figures and images for: Enhancement of HIFU thermal therapy in perfused tissue models using micron-sized FTAC-stabilized PFOB-core endovascular sonosensitizers
Source: Int J Hyperthermia. 2020 Sep 29;37(1):1116–30. doi: 10.1080/02656736.2020.1817575 (PMC8352380; doi:10.1080/02656736.2020.1817575)

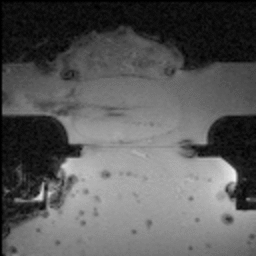

Supplement: Boiling Core Animation [file IHYT_A_1817575_SM2391.gif]
